# Interest of integrins targeting in glioblastoma according to tumor heterogeneity and cancer stem cell paradigm: an update

**DOI:** 10.18632/oncotarget.20372

**Published:** 2017-08-21

**Authors:** Laure Malric, Sylvie Monferran, Julia Gilhodes, Sabrina Boyrie, Perrine Dahan, Nicolas Skuli, Julie Sesen, Thomas Filleron, Aline Kowalski-Chauvel, Elizabeth Cohen-Jonathan Moyal, Christine Toulas, Anthony Lemarié

**Affiliations:** ^1^ INSERM U1037, Center for Cancer Research of Toulouse, Toulouse, France; ^2^ Faculty of Pharmaceutical Sciences, University of Toulouse III Paul Sabatier, Toulouse, France; ^3^ Department of Biostatistics, IUCT-Oncopole, Toulouse, France; ^4^ Department of Neurosurgery, Johns Hopkins University, Baltimore, Maryland, USA; ^5^ Department of Radiotherapy, IUCT-Oncopole, Toulouse, France; ^6^ Laboratory of Oncogenetic, IUCT-Oncopole, Toulouse, France

**Keywords:** glioblastoma, integrins, cancer stem cells, targeted therapy, clinical trials

## Abstract

Glioblastomas are malignant brain tumors with dismal prognosis despite standard treatment with surgery and radio/chemotherapy. These tumors are defined by an important cellular heterogeneity and notably contain a particular subpopulation of Glioblastoma-initiating cells, which recapitulate the heterogeneity of the original Glioblastoma. In order to classify these heterogeneous tumors, genomic profiling has also been undertaken to classify these heterogeneous tumors into several subtypes. Current research focuses on developing therapies, which could take into account this cellular and genomic heterogeneity. Among these targets, integrins are the subject of numerous studies since these extracellular matrix transmembrane receptors notably controls tumor invasion and progression. Moreover, some of these integrins are considered as membrane markers for the Glioblastoma-initiating cells subpopulation. We reviewed here integrin expression according to glioblastoma molecular subtypes and cell heterogeneity. We discussed their roles in glioblastoma invasion, angiogenesis, therapeutic resistance, stemness and microenvironment modulations, and provide an overview of clinical trials investigating integrins in glioblastomas. This review highlights that specific integrins could be identified as selective glioblastoma cells markers and that their targeting represents new diagnostic and/or therapeutic strategies.

## INTRODUCTION

### Glioblastoma

Glioblastomas (GB), classified by the World Health Organization as Grade IV-Diffuse Glioma [1], are the most frequent and lethal malignant primary adult brain tumor [2]. The current standard of care for newly diagnosed - or *de novo* - GB (∼90–95% of GB) includes maximal surgical resection and fractionated radiotherapy (30 × 2 Gy) with concomitant Temozolomide, also called the Stupp regimen [3]. However, prognosis remains extremely poor, with a median overall survival (OS) of 14–15 months [2]. A major molecular prognostic factor identified in GB is IDH1/2 mutations, a benefic prognosis factor that closely concerns secondary GB, which progress from low-grade diffuse astrocytoma or anaplastic astrocytoma (∼5–10% of GB) [1]. Another well-identified prognosis factor is the methylation status of the O^6^-alkylguanine DNA methyltransferase (MGMT) gene, encoding a DNA-repair enzyme for Temozolomide lesions. According to studies, ∼35–45% of wild-type IDH GB present a *MGMT* promoter methylation, associated to a better prognosis [4]. Recent studies have also highlighted new prognosis factors in GB, such as *TERT* promoter mutations (∼70–75% of *de novo* GB, worse prognosis factor), histone *H3F3A* K27 and G34 mutations (∼5% of adult GB), *ATRX* mutations and a positive glioma-CpG island methylator phenotype (G-CIMP), a benefic prognosis factor closely associated to secondary IDH mutant GB [4, 5]. *TP53* mutations (observed in 27% and 81% of IDH-wild type and IDH-mutant GB, respectively [1]) and *EGFR* amplification (∼40–50% of GB) and/or mutations, such as EGFR variant III, appear to be quite frequent in GB but do not seem to be associated to a worse outcome in GB patients [6]. Besides these molecular considerations, prompt relapses experienced by patients may be explained by the aggressiveness of GB, prone to invade surrounding brain tissue [2]. GB are also highly angiogenic, radio/chemoresistant and characterized by a strong cellular heterogeneity. Notably, a cancer cell subpopulation, called GB-initiating cells (GIC) or stem-like cells, appears to be particularly responsible for tumor maintenance and recurrence, as they can recapitulate the heterogeneity of the original brain tumor in orthotopically-xenografted nude mice [7].

**Figure 1:**
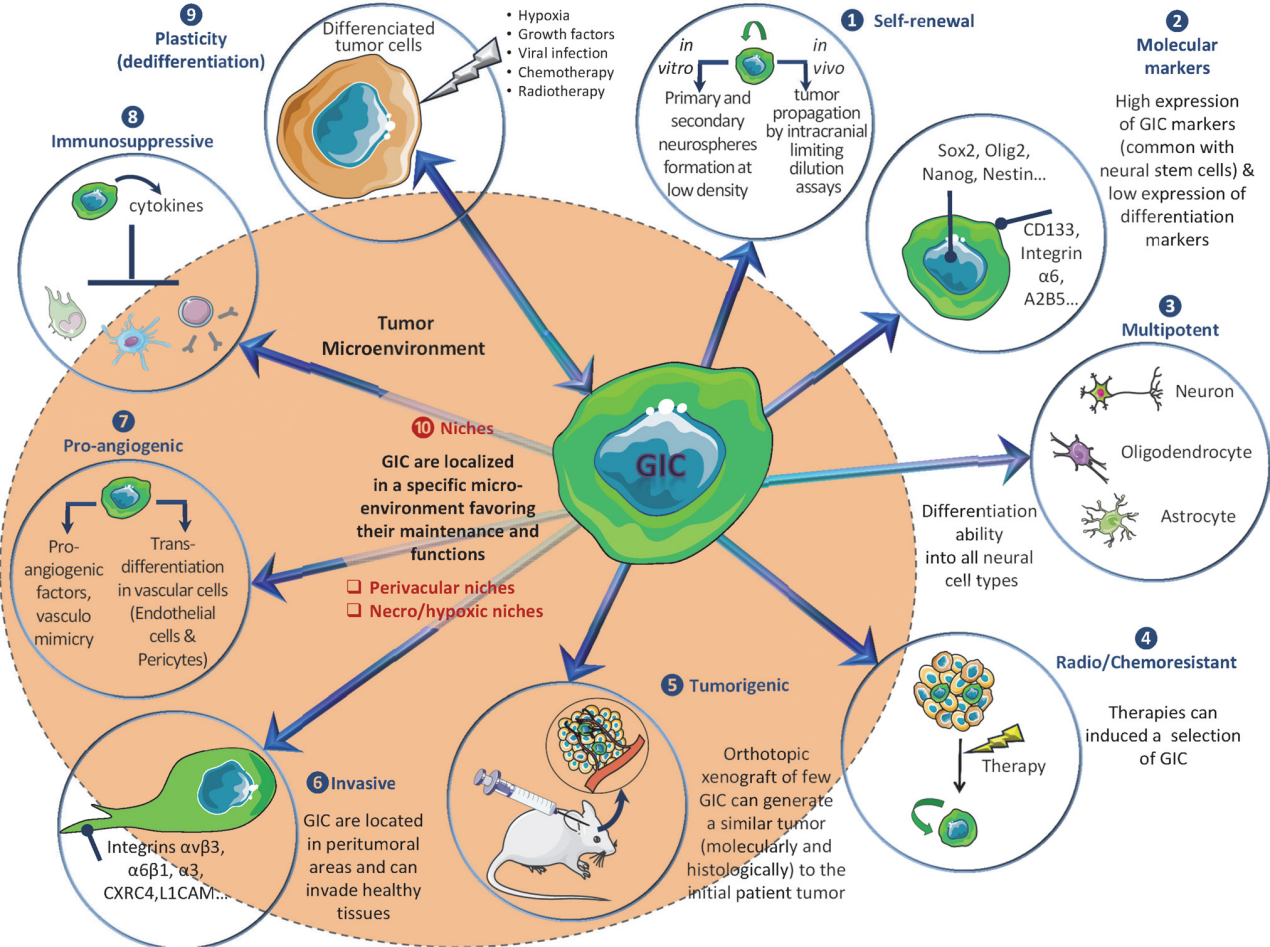
Overview of main GIC properties

GIC are characterized by their ability to self-renew *in vitro* (as neurosphere 3D structures) and *in vivo*, their marked expression of stem markers (CD133, Nestin, Olig2, Sox2, Nanog, BMI1, A2B5, ITGA6, L1CAM…), their multipotent aptitude to differentiate into neuronal, astrocytic or oligodendrocytic lineages, and a higher tumorigenic potential in orthotopically xenografted athymic nude mice [7]. Of note, it appears that the GIC subpopulation is strongly heterogeneous and could not be defined by the expression of an unique marker, but rather by a combination of markers. For example, it was previously shown that CD133-negative GB cells could also be identified as tumorigenic GIC [8]. Furthermore, GIC are particularly aggressive, invasive, radio/chemoresistant [7, 9] and display high autophagic capacities that may participate to these resistance processes [10]. GIC participate to angiogenesis via pro-angiogenic factor synthesis, cooperation with endothelial cells (EC), vasculogenesis by vasculogenic mimicry formation and transdifferentiation into pericytes or EC (for review [11]). GIC can also induce immunosuppression, notably via cytokines secretion [12]. Moreover, several signals and stresses, such as hypoxia [13], specific growth factors [14, 15], viral infections [16], chemotherapy [17] and ionizing radiations [9] contribute to the generation/amplification of GIC population from more differentiated GB cells, a process, which can evoke a possible cancer cell dedifferentiation or reprogramming [18]. Collectively these properties (Figure 1) suggest that targeting GIC represents a therapeutic interest in GB. To this end, blockade of specific pathways identified in GIC maintenance and functions (Notch, Sonic Hedgehog/Wnt, Akt…) may be a valuable strategy [7], but could lack efficiency due to compensatory pathways. Another alternative to eradicate GIC could be the targeting of their niches. Actually, GIC preferentially reside in perivascular and necro/hypoxic niches where they closely interact with the microenvironment [19]. These interactions with microenvironment elements, like stromal cells or extracellular matrix (ECM) components, seem to be critical for GIC maintenance, notably through metabolic and/or epigenetic modifications [19, 20] and could constitute a putative target.

### Integrins

**Figure 2:**
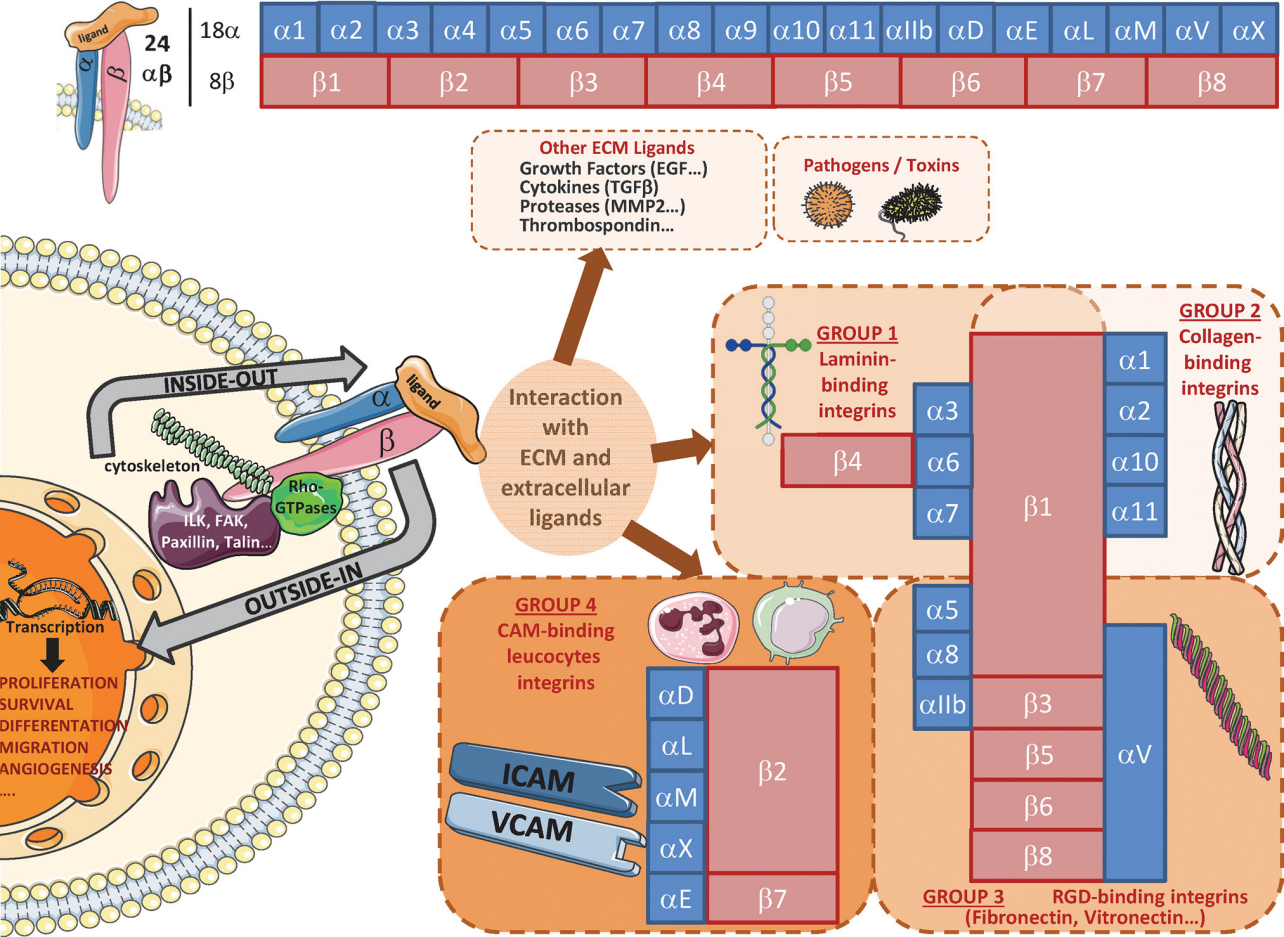
Overview of main integrin heterodimers and ligands

Key components of the dialogue between cells and the microenvironment, integrins (ITG) are composed of two non-covalently associated α and β subunits, which are characterized by a large extracellular domain, a short transmembrane domain and a small intracellular non-catalytic cytoplasmic tail [21]. To date, 18 α and 8 β subunits have been identified in humans and form at least 24 unique heterodimers. Each α/β combination determines specific integrin binding ability and functions. Structural characteristics and ligands of these heterodimeric glycoproteins have already been reviewed [22] and are summarized in Figure 2. These receptors play a role in the regulation of cell adhesion to ECM proteins or cell surface proteins (immune cells, platelets…) [22]. They are central regulators, which act as transmembrane links between extracellular contacts and intracellular cytoskeleton via a bidirectional signalling. First, upon extracellular ligands binding, integrins cluster in the membrane and transduce intracellular signals through their cytoplasmic domain (mostly via β subunit) by activation of kinases (Focal Adhesion Kinase (FAK), Integrin-Linked Kinase (ILK)…) or Rho-GTPases. Integrins can then activate pathways leading to gene transcription to sustain proliferation, survival, differentiation and migration (outside-in signalling) [22, 23]. Second, cytoplasmic proteins can also modulate integrins extracellular affinity for their ligands (inside-out signalling). In addition, integrins also bind cytoskeleton proteins (α-actinin, tensin, vinculin, talin, paxillin, intermediate filaments…) and, then, are involved in structural cell functions. All these characteristics (Figure 2) may confer a high degree of complexity and flexibility in integrin-linked cell functions and signalling pathways, since several distinct integrins can be expressed on specific cells and, depending on context generate dramatically different responses. As a consequence, integrin-pathways alterations have also been linked to several pathologies, such as auto-immune and thrombotic diseases, ischemic brain injury, inflammation, fibrosis and cancer.

### Integrins in cancer

Integrins are rarely mutated in tumors. They do not act as oncogenes but may cooperate with oncogenes to favor tumorigenesis, either by particular expression and/or localization in tumor cells compared to normal cells, by post-translational modifications or via integrin recycling. Indeed, in several cancers, modifications of the integrin pattern are often associated with tumor progression. For example, α6β4, α5β1, αvβ6, and αvβ3 are upregulated in different tumor types, including GB [24, 25], and correlated with poor patient survival. However, integrin expression can also be decreased in tumors (e.g. α2β1 in breast cancer, α6β4/α6β1 in esophageal carcinoma), but still in favor of cancer progression [23, 24]. Altered integrins expression has also been reported in different models of carcinogenesis induction [24]. Moreover, post-translational modifications (glycosylation/sialylation, citrullination and carbamylation) modulate integrin conformation, affinity and functions and play a role in cancer progression [26, 27]. Finally, increased integrin recycling is associated with invasive and metastatic processes in cancer cells [28].

All these modulations affect integrins functions. First they are involved in invasion (and metastasis) as they directly provide the traction necessary for cell migration (for review [24]). They also participate in ECM remodeling through protease regulation and localization at migration front. Subsequently, integrins regulate tumor cell proliferation and survival through ECM-direct binding and/or intracellular signalling [24]. Furthermore, integrins interact and crosstalk with other cell surface receptors, like growth factor receptors (for review [29]). Finally, integrins are expressed by various tumor microenvironment cells (EC, pericytes, fibroblasts, immune cells, platelets…) and allow cell interactions to promote tumorigenesis. Integrin involvement in angiogenesis is particularly well described [30], as well as their role in epithelial to mesenchymal transition (EMT) which often mediates the acquisition of a migratory and/or invasive phenotype in cancer cells. Even if GB cannot be considered as tumors of epithelial origin, EMT can occur in response to treatment [31]. For example, α5β1 integrin was shown to mediate EMT in GB cells [32]. Consequently, integrins contribute to cancer progression and future studies have to identify the roles of these regulators precisely.

Due to their involvement in tumor cell functions, their crosstalk with numerous pathways and their localization at the cell membrane, integrins represent promising targets for cancer treatment. Consequently, different integrin inhibitors are under clinical investigations, notably in GB (detailed below). However, disappointing results in a phase III-study [33] forced researchers to reappraise the roles of the different integrins in GB, notably their specific expression according to tumor heterogeneity. In this review, we summarized integrin expression patterns and characteristics in GB and particularly concentrated on tumor molecular and cellular heterogeneity, notably regarding GIC. We chose to specifically focus on newly diagnosed GB and to not address here lower grades diffuse gliomas. We also discussed the roles of these adhesion molecules in GB progression and made an overview of therapeutic options.

## ROLES OF INTEGRINS IN GLIOBLASTOMA

### Integrins expression in glioblastoma

αvβ3 and αvβ5 were first identified as attractive therapeutic targets in GB [34, 35]. These integrins are not expressed on normal brain cells [35] but specifically on both tumor-associated EC and GB cells [35]. Their ECM ligands, vitronectin [34] and fibronectin [36], are also upregulated in GB. Currently, αvβ3/αvβ5 and α6β1, a laminin-interacting integrin also overexpressed in GB [37], are the most studied in GB but others may play major roles. Indeed, comparative immunohistochemistry (IHC) staining of integrins in GB versus control tissues reveal an overexpression of α2, α3, α4, α5, α6 and β1 [38], as well as of β3/αvβ3, αvβ5 and αvβ8 [39]. Of note, β8 and α5β1 were particularly observed in peri-necrotic and perivascular areas [40].

**Figure 3:**
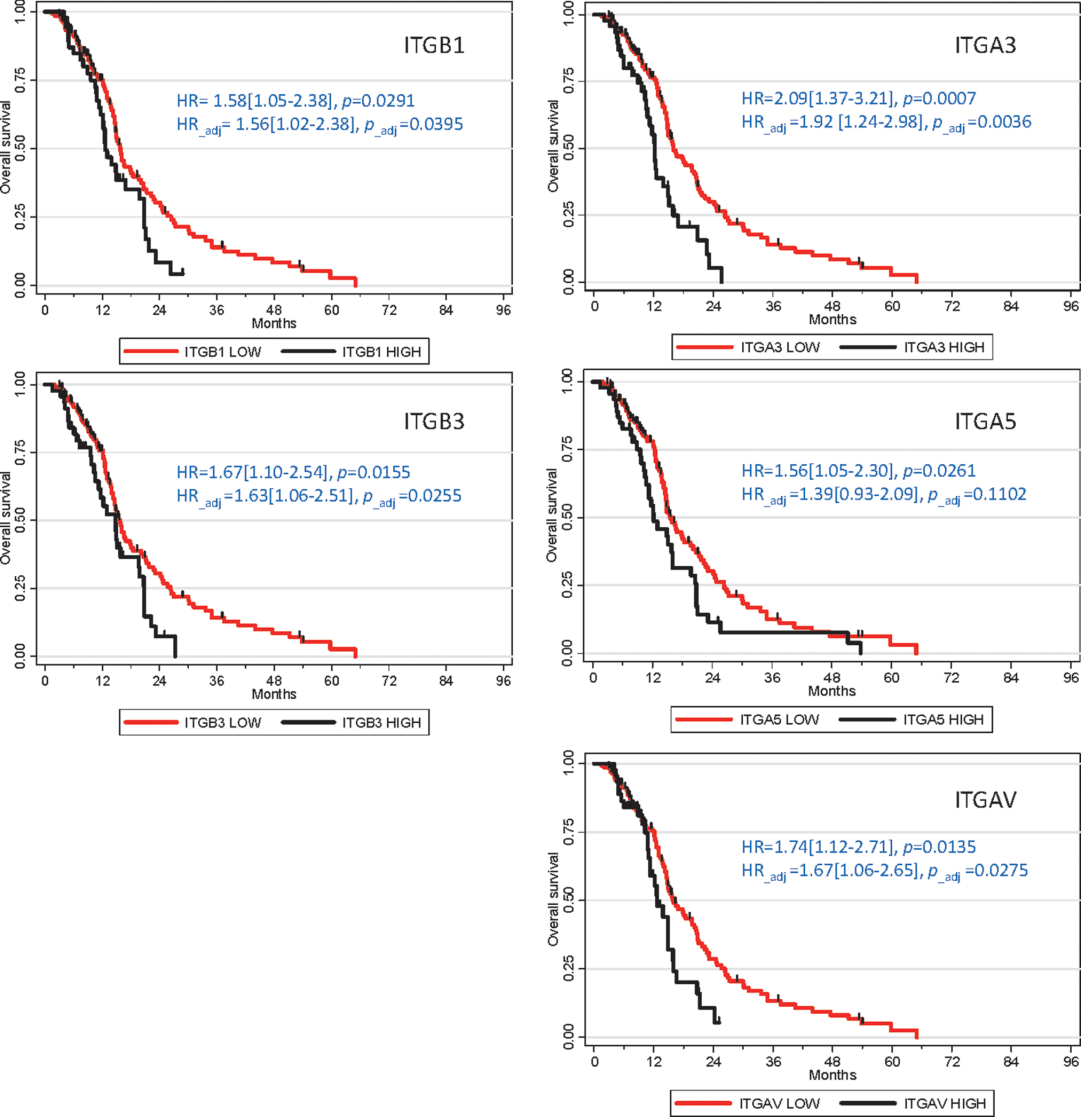
Integrins overexpression association with poor prognosis in GB patients Kaplan-Meier survival plots were established using TCGA Affymetrix dataset (*n* = 184). Hazard ratios (HR) and *p*-values were given for univariate analyses. Adjusted HR and *p*-values were calculated for multivariate analyses, in relation to other prognostic clinical covariates (Age, G-CIMP status and Karnofsky score).

Although several integrins are overexpressed in GB, links between integrin expression and patient overall survival (OS) were scarcely explored. α3, α5, α7, αvβ3, αvβ5 and αvβ8 expression levels were positively associated with higher glioma grades [35, 39, 41–43]. αvβ3 or α3β1 overexpressions at the protein level were also correlated with poor GB prognosis [25, 43]. Consequently we conducted a statistical analysis (detailed in supplemental data) based on TCGA Affymetrix data [44] to determine GB patient OS according to major integrin expression. We focused on a group of 184 newly diagnosed primary GB solely treated with standard chemoradiotherapy [3]. In this particular population, overexpression (based on an upper threshold of the third quartile) of β1, β3, α3, α5 and αv, but neither β5, β4, α6 nor α7, was significantly associated with OS decrease, whether through univariate or multivariate analysis (Figure 3). α5 and α3 overexpressions were previously associated to worse prognosis in GB (*n* = 127, TCGA dataset and high-grade glioma cohorts, for α5 [42, 45]) or grade III/IV glioma patients (*n* = 68, immunohistochemistry data, for α3 [43]). It was also recently showed that β1 may be downregulated at both protein and RNA levels in GB patients with OS > 23 months (*n* = 14/26) [46]. However, α6, hypothesized to be associated with decreased OS in all-grade glioma [47] and in three-fold (or more) overexpressing GB (*n* = 7/193 patients, REMBRANDT dataset [48]), fails to show a similar pattern in our analysis. Similarly, no difference could be highlighted for β4, shown to be associated with GB worse prognosis (*n* = 393, TCGA dataset) [49]. Lower expression of α7 integrin, which was recently identified as a new functional marker in GB (probably as a heterodimer with β1), was also correlated with better prognosis outcomes in TCGA GB patients (*n* = 595) and in three additional independent GB cohorts [41]. However, our analysis failed to highlight such survival gain in α7-low patients. These disparities for α6, α7 and β4 could be linked to the fact that we restrained our analysis to a homogenous primary GB patients subgroup treated with standard chemoradiation.

Whereas several works studied the expression of integrins at the RNA level in GB patient cohorts, data of their expression at the protein level are scarce in such cohorts. CORE (*n* = 224) and CENTRIC (*n* = 274) clinical trial cohorts were explored for αvβ3, αvβ5 and αvβ8 staining by IHC and showed that αvβ3 is mainly expressed by GB endothelial cells, contrary to αvβ8 which is expressed almost exclusively by GB tumor cells. Of note, αvβ5 is expressed by both cell populations [50]. These results confirm those found in an independent cohort of 324 patients for which 147∼160 GB samples were stained by IHC [39]. Proteomic analyses also demonstrated that αv integrins are overexpressed in GB endothelial cells compared to physiological endothelial cells (10 GB samples) [51] and that sialylated β8 integrin is upregulated in GB samples in comparison with control adult astrocytes [27]. Using the Human Protein Atlas resource portal [52], we also noticed that α3, αv, β1, β4, β5 and β8 integrins may be overexpressed in high grade glioma patient samples compared to control cortex tissues (IHC data), confirming previous studies [38, 39, 43].

These data show that several integrins are overexpressed in GB and are associated with worse GB prognosis, indicating that they could play a role in GB progression and that specific targeting of overexpressed-integrins could be of therapeutic interest in GB.

### Integrins and glioblastoma molecular subtypes

Several integrins are overexpressed in GB [35, 38–40, 42, 43, 49] but these results have to be analyzed in the light of GB molecular heterogeneity. Indeed, based on RNA and genomic profiling using TCGA databases, GB were classified by Verhaak et al. in four subtypes: Pro-Neural, Neural, Classical and Mesenchymal [53]. Philips et al. also classified GB of a different dataset in three subtypes (Pro-Neural, Proliferative and Mesenchymal), with Proliferative GB corresponding to TCGA Neural and Classic groups (40–50% of GB). In addition to this genomic heterogeneity between GB patients, it was also demonstrated that intratumoral heterogeneity, with all four subtypes being represented, can occur within a same GB tumor area, either between spatially distinct fragments [54] or even at the single-cell level [55]. Each subtype is characterized by specific genetic alterations. The Proliferative signature is characterized by EGFR amplification and stem marker overexpression (Nestin, Notch and Hedgehog). Within this group, it was originally described that Neural GB overexpress neuronal markers (e.g. NEFL). Pro-Neural subtype (5–20%) is associated with TP53 mutation, PI3K-pathway overactivation, PDGFRa amplification and Olig2/Sox2 stem markers overexpression. A small IDH1-mutants subpopulation was also exclusively found in Pro-Neural GB. Mesenchymal subtype (35–50%) is characterized by several deregulated pathways (TNF/NFκB, MET, YKL40, CXCR4, TGF-β, CD44…), a very invasive and angiogenic phenotype and a strong aggressiveness [56]. Interestingly, single-cell analysis demonstrated that a notable stemness signature was associated to Pro-Neural and Classic cell subtypes, and underrepresented in the Mesenchymal subtype [55]. In line with this, Mesenchymal GB were shown to contain a more progenitor type of GIC, contrary to Pro-Neural tumors containing a more stem GIC type [56, 57]. Of note, major studies recently classified GB by combining RNA, genomic and DNA methylation profiles [58, 59]. This new classification into six different clusters (LGm1 to LGm6) showed that primary GB (IDH wild type) can be classified into three subsets, with LGm4 being enriched in Classic-like GB, LGm5 in Mesenchymal-like GB and LGm6 in Pro-neural and Mesenchymal GB [59]. The Neural group, although represented in those three subsets, failed to show any specificity for any of these new subtypes. Finally, the newest study of the Verhaak group was designed to characterize the transcriptional heterogeneity of *IDH* wild-type GB by only using genes solely expressed by tumor cells and not by tumor-associated cells [60]. For this purpose, they combined RNA-sequencing data of (i) ∼600 single cells isolated from 8 GB, (ii) 37 paired GB bulk tumors and their *in vitro* derived-neurospheres and (iii) matching microdissected tissues of both GB tumor cores and leading edges. They concluded that *IDH* wild-type GB can be classified into three subgroups (Pro-neural, Classic and Mesenchymal) and that the previously described Neural phenotype could be non-tumor specific [60].

**Figure 4:**
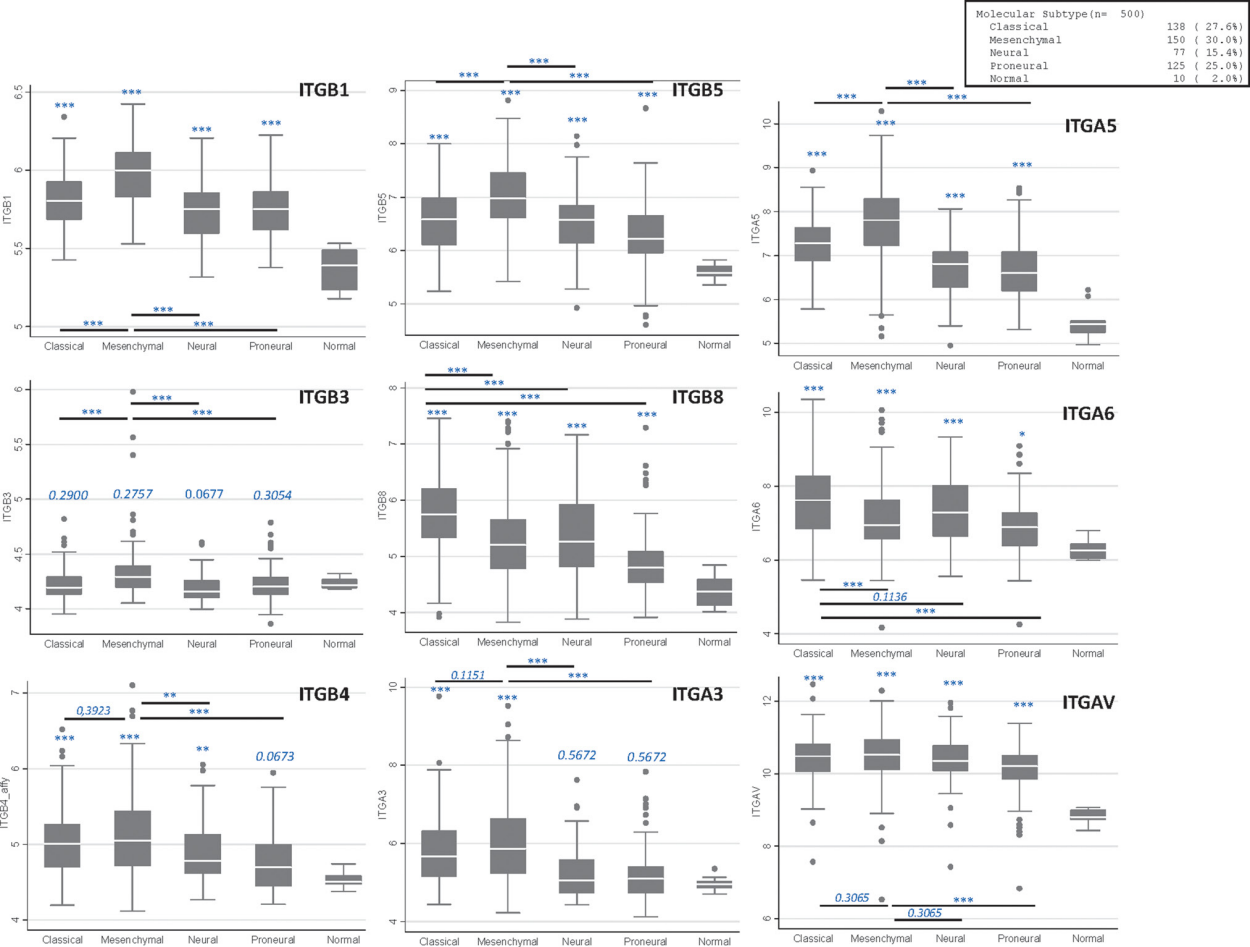
Main integrins expression in GB samples according to molecular subtypes Plots were established using TCGA Affymetrix dataset (*n* = 500). *p ≤ 0.05; ** *p* ≤ 0.01; ***p ≤ 0.001. When not stated, values are compared to the “normal” subgroup.

Regarding integrin, very little was known, to our knowledge, about their differential expression among the different GB subtypes, neither in the different subsets defined by RNA profiling nor in the new LGm clusters. We then used publically available TCGA data [44] to performed a statistical analysis (see supplemental data) of the major integrins expression in all primary GB (*n*=500) and showed that most of them (β1, β4, β5, β8, α3, α5, α6, α7, αv) are significantly overexpressed in GB compared to normal brain (Figure 4, Supplementary Figure 1 and Supplementary Table 1A). However, contrary to numerous studies showing β3 transcript overexpression in GB [35, 39], this particular integrin did not appear to be overexpressed in GB for this specific dataset. We then analyzed an additional TCGA dataset, based on RNA-seq profiling of *n* = 149 primary GB and found that β3 is indeed overexpressed in GB (Supplementary Table 1B), as in the REMBRANDT dataset [39]. In addition, all these integrins but three (β8, α6 and α7) show a stronger overexpression in Mesenchymal GB compared to other subtypes and a lower overexpression in Pro-Neural GB (β3 excepted) (Figure 4 and Supplementary Figure 2). However, β8, α6 and α7 display a distinct pattern, with a higher overexpression in Classic GB compared to Mesenchymal and Pro-Neural GB (Figure 4), confirming some recent data on α7 [41]. In line with this, α7 overexpression was shown to especially correlate with poor prognosis outcome in Pro-neural GB patients (TCGA dataset) [41]. In support to our analysis, *ITGB8* and *ITGA7* were previously described as genes related to the Classic subtype signature [55]. Moreover, *ITGB1* was defined as a top gene of the Mesenchymal subtype signature [60]. Of note, the last study by Verhaak’s group highlighted that Mesenchymal GB are enriched in tumor associated microglial cells, as the macrophage marker *ITGAM* (CD11b) appeared, among others, to be specifically overexpressed in this subtype [60].

According to our analyses, two different integrin expression patterns were then highlighted according to GB subtypes: Mesenchymal GB show a global integrin overexpression compared to other subtypes, apart from β8 and α6, mainly overexpressed in Classic GB. These specificities could be of interest in clinic to target selective integrins according to their overexpression in particular GB subtypes.

As an additional and relevant critical aspect of GB heterogeneity in clinic, it would be of major interest to decipher whether or not integrin expression profile could vary over time between primary GB and the associated recurrent tumors. Several studies were recently undertaken to assess this longitudinal transcriptomic heterogeneity in GB [60, 61]. It is noteworthy that the genomic and transcriptomic analyses of recurrent tumors revealed in these GB the presence of some exclusive LTBP4 (latent TGF-β binding protein 4) mutations associated to its overexpression [61]. As further highlighted in the present review, LTBPs, some key components of the latent TGFβ complex, appeared to be required for TGFβ activation in response to the binding of specific αv integrins (mainly αvβ6 and αvβ8) to the latent TGFβ complex. So, recurrent GB may present a deregulation of the TGFβ pathway, known to sustain GB aggressiveness, and it would be of interest to explore the role of the related αv integrins in this deregulation.

### Integrins in glioblastoma proliferation, migration and invasion

Integrins sustain tumor cells proliferation, notably via their association with ECM components and maintenance of cell adhesion to substrate [24]. Few studies were conducted in GB. α5β1, which reduces GB cell proliferation when inhibited [62], acts through its interaction with Anosmin-1 [63] or Rap1A Rho-GTPase [64]. β4 interaction with its extracellular ligand netrin-4 also sustained GB cell proliferation [49].

However, integrins contribution to adhesive, migratory and invasive behaviors has been extensively studied in GB. First, αvβ3/αvβ5 encourage GB cell migration/invasion by direct adhesion to ECM (via fibronectin, vitronectin, osteopontin or periostin) [36, 65, 66] and then activate intracellular signalling pathways. For example, they trigger FAK, which controls cytoskeletal organization, force generation and survival, or activate additional pathways (Shc/MAP-Kinases, Rho-GTPases, Src Family Kinases) [23, 24, 67]. αvβ3 promotes GB invasion via the activation of MMP-2 at the plasma membrane and the subsequent degradation of complex ECM [24]. Finally, αvβ3/αvβ5 inhibition in murine models reduces GB cell migration/invasion [68]. Besides αvβ3/αvβ5, α6β1 was also associated with a stronger invasive phenotype. Its expression in U87 GB cell line enhances cells invasion *in vitro* and *in vivo* [37], as well as in GIC (our unpublished data). α6β1 cooperation with ERK and N-cadherin could support this effect [69]. Recently, β1 (associated with α3, α5 or α9) [45, 62, 70–72] and β8 [73, 74] have also been involved in GB cells migration/invasion, even if mechanisms are not well known yet. α5β1 could act through its interaction with MMP-2 [75] and αvβ8 through the modulation of Rho-GTPases activities (via RhoGDI1) [73].

Consequently, targeting specific integrins in GB could reduce tumor invasion and aggressiveness.

### Integrins in glioblastoma angiogenesis

Knockout-strategies demonstrated the role of integrins in tumor angiogenesis [30]. In GB, characterized by a high vascularization and an overactive angiogenesis depending on VEGF and bFGF [76], this process is notably mediated by αvβ3, αvβ5, β1 and αvβ8. EC-expressed αvβ3/αvβ5 can provide survival signals and traction for invading cells, two mechanisms necessary to angiogenesis [34, 35]. αvβ3- and αvβ5-associated angiogenesis are respectively dependent on tumor cell-secreted bFGF/TNFα and VEGF through an amplification loop leading to αvβ3/αvβ5 overexpression on EC [77]. Overexpressed αvβ3/αvβ5 mediate in turn adhesive interactions with ECM proteins (vitronectin, fibronectin, fibrinogen, osteopontin, von Willebrand factor). In cooperation with bFGF/VEGF, αvβ3/αvβ5 also activate signalling pathways (FAK/ILK, PI3K/Akt and SDF1-CXCR4 [78]) that promote EC proliferation, survival and migration [30]. Regarding PI3K/Akt, a recent study associates this pathway with Plexin-B1/Rho/αvβ3 and Serine/arginine Protein Kinase-1 [79]. Moreover, migration can be improved by αvβ3 interaction with pleiotrophin (a secreted heparin-binding cytokine) on GB cells and EC [80] and with the cell adhesion molecule L1CAM on GIC [81]. Besides αvβ3/αvβ5, β1 is also involved in GB angiogenesis [76]. α5β1, similarly to αvβ3, is also upregulated by FGF on tumor endothelium during angiogenesis and promotes EC survival and migration [82]. β1 can be associated with other α-subunits, like α9, to support EC-mediated angiogenesis in GB [83]. α4β1 is also involved in pericyte-dependent angiogenesis to favor blood vessel maturation/stabilization [30]. Finally, αvβ8, overexpressed on GB cells [35, 40, 74], is involved in vessel formation/remodeling in GB through an autocrine TGF-β-dependent differential control of angiogenesis [74].

To summarize, several integrins, notably the αv and β1 families, appear to be interesting targets in GB to reduce angiogenesis in these highly vascularized brain tumors.

### Integrins in glioblastoma survival and resistance to therapy

Depending on the context, integrins can either enhance cell survival or apoptosis, as they constantly challenge their microenvironment to regulate the death/survival balance. However, cancer cells deregulate this balance to promote survival. These different mechanisms, i.e. integrin ligation-related activation of pro-survival signals and unligated-integrin effects on both Integrin Mediated cell Death (IMD) or Anoikis (cell detachment-induced apoptotic death) were previously reviewed [24]. Briefly, IMD, a Caspase 8-dependent apoptotic process activated by unligated-integrins [24], can be deficient in tumor cells to promote survival [84]. In glioma, integrin inhibition can also induce a TGFβ-dependent anoikis [85]. Finally, in GB, both αvβ3 and the cytoskeleton regulatory kinase PAK4 were recently highlighted to mediate evasion of a p21-dependent senescence [86].

Moreover, chemo/radiotherapy exposure was associated with alterations of integrin-linked pathways in GB. First, irradiation up-regulated αvβ3 in U87 cells [87]. Second, α2β1, α3β1 and α5β1 were overexpressed in multi-drug resistant human glioma cells [88]. These results suggest, as for other cancers, an important role of integrins in GB resistance after chemo/radiotherapy. Cell adhesion to ECM, particularly through integrin ligation, is known to confer resistance in tumors, either to chemotherapy (cell-adhesion-mediated drug resistance, CAM-DR), or to irradiation (cell-adhesion-mediated radioresistance, CAM-RR).

Regarding CAM-DR, β1 and β4 integrins can confer Temozolomide-resistance in GB cells [89, 90]. An α5β1/p53 interconnection was notably demonstrated to modulate two anti-apoptotic proteins: Phosphoprotein enriched in astrocytes 15 (PEA-15) and Survivin. α5β1-overexpressing GB may then benefit from targeted-therapies associating integrin antagonists and survivin repressors [91]. Another putative and more general mechanism could be through integrin interaction with Periostin, an ECM protein recently highlighted in anti-angiogenic therapy resistance in glioma models [92]. Finally, as the high GB intratumoral heterogeneity (depicted above and in [54, 55]) could have major clinical implications in resistance to treatments, it is noteworthy that a recent single-cell clonal analysis of a GB patient sample by RNA-sequencing highlighted that TMZ-resistant clones displayed an up-regulation of αv integrin and a downregulation of α7 integrin compared to TMZ-sensitive clones [93].

CAM-RR is more documented in GB. U87 and SF763 GB cells, which express αvβ3 and αvβ5, respectively, were shown to be radioresistant through an ILK/RhoB pathway. Specific αvβ3/αvβ5 inhibition by Cilengitide or ILK blockade led to a significant *in vitro* radiosensitization [94]. *In vivo*, U251 cells-xenografted rats treated with Cilengitide and radiotherapy displayed a survival advantage compared to radiotherapy alone [95]. This radiosensitization was associated with an increase of radiation-induced apoptosis [95]. Furthermore, EC radiosensitization can also induce GB cell radiosensitization *in vitro* and *in vivo* [95]. So αvβ3/αvβ5 blockade could lead to radiosensitization potentiation by targeting both endothelial and tumor cells. Another integrin involved in GB CAM-RR is β1, through the activation of a pro-survival pathway transduced by Akt, paxillin, p130Cas and JNK [96]. β1 and JNK co-inhibition was recently shown to potently radiosensitize GB-initiating cells *in vitro* and *in vivo* [97]. ERK may also be involved since this MAP-kinase participates to chemoresistance in glioma cells [89]. Then, several studies imply integrins in GB radioresistance. Growing evidence demonstrated that induction of survival pathways (PI3K/Akt, NFKB, Bcl2, JNK…) or inhibition of pro-apoptotic pathways (p53), most often through FAK activation, could be involved [23, 98, 99]. The anti-apoptotic survivin, induced by irradiation [100], can also modulate the radiotherapy-induced mitotic U87 cell death through an ILK/HIF-1α/survivin pathway [101]. Besides ILK/FAK, other focal adhesion proteins such as PINCH1 and ILKAP contribute to GB radioresistance [102]. Among potential mechanisms in GB, integrins could also act by cell cycle modulation [103], since irradiation-induced cell cycle arrest is potentiated by α6β4/laminin-5 adhesion in prostate cancer [104]. Moreover, IMD resistance might also occur after radiotherapy. Indeed, as irradiation induces adhesion of breast cancer cells to laminin, fibronectin and collagen [105], it could be hypothesized that such process impairs IMD. Finally, integrins cooperation with growth factor pathways could also mediate CAM-RR in GB, as concurrent inhibition of β1/EGFR can radiosensitize tumor cells [106].

Altogether, these observations suggest that several integrins, particularly αvβ3/αvβ5/β1/β4, are interesting candidates in GB to be combined to ionizing radiations for radiosensitizing strategies. However, most of these studies were conducted on adherent GB cell lines and this could limit the significance of these resistance pathways in clinic. To better recapitulate the behavior of GB cells *in vivo*, other *in vitro* GB models could be more appropriate, and notably the GIC-enriched neurosphere 3D cell model.

### Integrins in glioblastoma-initiating cells

Integrins were recently highlighted in cancer stem cell (CSC) biology, as they could represent specific CSC biomarkers and/or participate to CSC phenotype and functions [107]. In GB, α6 was shown to be preferentially and strongly expressed by GIC compared to differentiated cells [48]. Furthermore, α6^High^-expressing GIC present higher proliferation, stronger neurospheres-forming ability *in vitro* and tumorigenesis *in vivo* compared to α6^Low^ GIC [48]. α6 also regulates GIC invasion process by cooperation with N-cadherin and ERK pathway [69]. α3 integrin was also shown to be overexpressed in GIC and to promote invasion [71]. Of note, the cell surface tetraspanin family member CD151 was recently found as a novel regulator of GIC by interacting with α6 and α3 [108]. Recently, a monoclonal antibody screening identified α7 integrin as a functional GIC marker. Indeed, α7 appeared to sustain the stem characteristics, the laminin-linked invasion ability and the tumorigenic capacity of this GIC subpopulation [41]. Besides these three α integrins, β1 integrin, even if not specifically expressed by GIC, can form a complex at the GIC membrane with astrocytes-secreted Connective Tissue Growth Factor and tyrosine kinase receptor type A and activate NFκB-driven invasion [109]. Another argument linking integrins with GIC biology is that particular integrins, belonging notably to the αv-family, can activate TGF-β pathway [110]. Indeed, TGF-β was described as a stemness gatekeeper in glioma [111] and TGF-β receptor antagonists target GIC and reduce tumorigenesis and radioresistance [112]. Finally, besides integrins, the junctional adhesion molecule-A may also promote GIC self-renewal [47].

In addition, recent studies also highlighted the differential expression of integrins in GIC according to their molecular subtypes. Indeed, even if molecular classifications were originally set up on GB whole tissues, GIC can also be classified according to their transcriptional subtypes. In that way, it was shown that GIC can be clustered in two major subgroups with distinct functional and molecular properties [56, 57, 113, 114]. The first one corresponds to a more stem-like GIC group, characterized by CD133 overexpression, an increased rate of asymmetric division, a higher invasive phenotype in xenografted-mice and a significant enrichment in Pro-Neural genes. The second group matches with a progenitor-like GIC type, corresponding to more adherent cells/neurospheres, a decrease in stem marker expression (Olig2, CD133), a trend toward a decreased tumorigenic ability in orthotopically-xenografted mice and an enrichment in Mesenchymal genes. These two subgroups were confirmed in two studies realized either in 48 different patient-derived GIC cell lines [115] or at the intratumoral level in a spectrum of GIC clones derived from the same tumor [116]. In the study by Xie and colleagues, an additional GIC cluster, corresponding to the Classic subtype, was highlighted, the Neural subtype being underrepresented (*n* = 3/48) [115]. Pro-Neural GIC clones or cell lines were found to be more sensitive to chemo- and radiotherapies, to have a higher proliferation rate and to give a higher rate of macroscopic tumors in xenografted mice [115, 116]. Regarding the correlation of GIC subtypes with survival prognosis in GB patients or in xenografted-mice, no clear trend could be defined since opposite results were obtained according to the studies, with a worse survival either for Mesenchymal GIC [56, 117] or for Pro-Neural GIC [115, 116].

This molecular and functional heterogeneity between GIC clones/cell lines was shown to be associated with differential integrin expression between GIC subgroups. Indeed, integrin α4 appeared to be downregulated in Pro-Neural stem-like GIC [114]. Integrins β1 and α5 were demonstrated to be overexpressed in Mesenchymal progenitor-like GIC at the transcriptional level [116, 117]. β1 overexpression was also confirmed at the protein level in Mesenchymal GIC clones [116], as well as the Mesenchymal marker CD44, known to bind and activate α5β1 in cancer cells [118]. This suggests an important role for α5β1 integrin in Mesenchymal GIC. Of note, this Mesenchymal GIC subtype is characterized by a down-regulation of miR-9-3p compared to Pro-Neural GIC [119]. As *ITGB1* is a known target inhibited by miR-9-3p in cancer cells [120], this could contribute to the overexpression of β1 in Mesenchymal GIC. Finally, we used the Human Glioblastoma Cell Culture (HGCC) resource portal (www.hgcc.se, detailed in [115]) to compare the expression of the main integrins in the 48 GIC cell lines according to their molecular subtypes. We noticed, as we observed in the TCGA dataset analysis detailed above, that integrins α3, α4, α5, αv, β1, β3, β4 and β5 were overexpressed in the Mesenchymal GIC compared to the Pro-Neural GIC cell lines. All these integrins, except β5, were also downregulated in the Classic GIC in comparison with Mesenchymal GIC. On the contrary, α6 failed to show any significant variations between the different subgroups. β8, as observed in the TCGA Affymetrix dataset, is overexpressed in the Classic GIC subtype compared to Pro-Neural/Mesenchymal GIC cell lines. Altogether, it can be postulated that most of the integrins with a role in GB are preferentially expressed in Mesenchymal GB cells and GIC compared to Classic/Pro-Neural subtypes, with the notable exception of α6 and β8, preferentially expressed in the Classic GB cells.

Besides this molecular heterogeneity between GIC populations, it was also shown that anatomical location of the biopsy has an impact on integrin expression in GIC. Indeed, GIC generated from the GB peritumoral area displayed a higher migratory and invasive phenotype compared to GIC from the tumor mass. This invasive capacity was correlated to the overexpression of integrin β3 in peritumoral GIC and inhibition of αvβ3 blocked this invasive phenotype [121].

To conclude, specific integrins, such as α6, seem to contribute to GIC identification and/or functions. Moreover, the GIC heterogeneity, either at the molecular or anatomical levels, between GB patients or even between GIC clones of a same patient, is associated with a differential integrin expression. Notably, β1 integrin displayed an important overexpression in Mesenchymal GIC subtypes. Consequently, targeting these specific integrins preferentially expressed in GIC or in certain GIC subtypes might represent a potent way to alter GIC stemness and tumorigenicity in order to improve GB treatment.

### Integrins in glioblastoma microenvironment and niches

**Figure 5:**
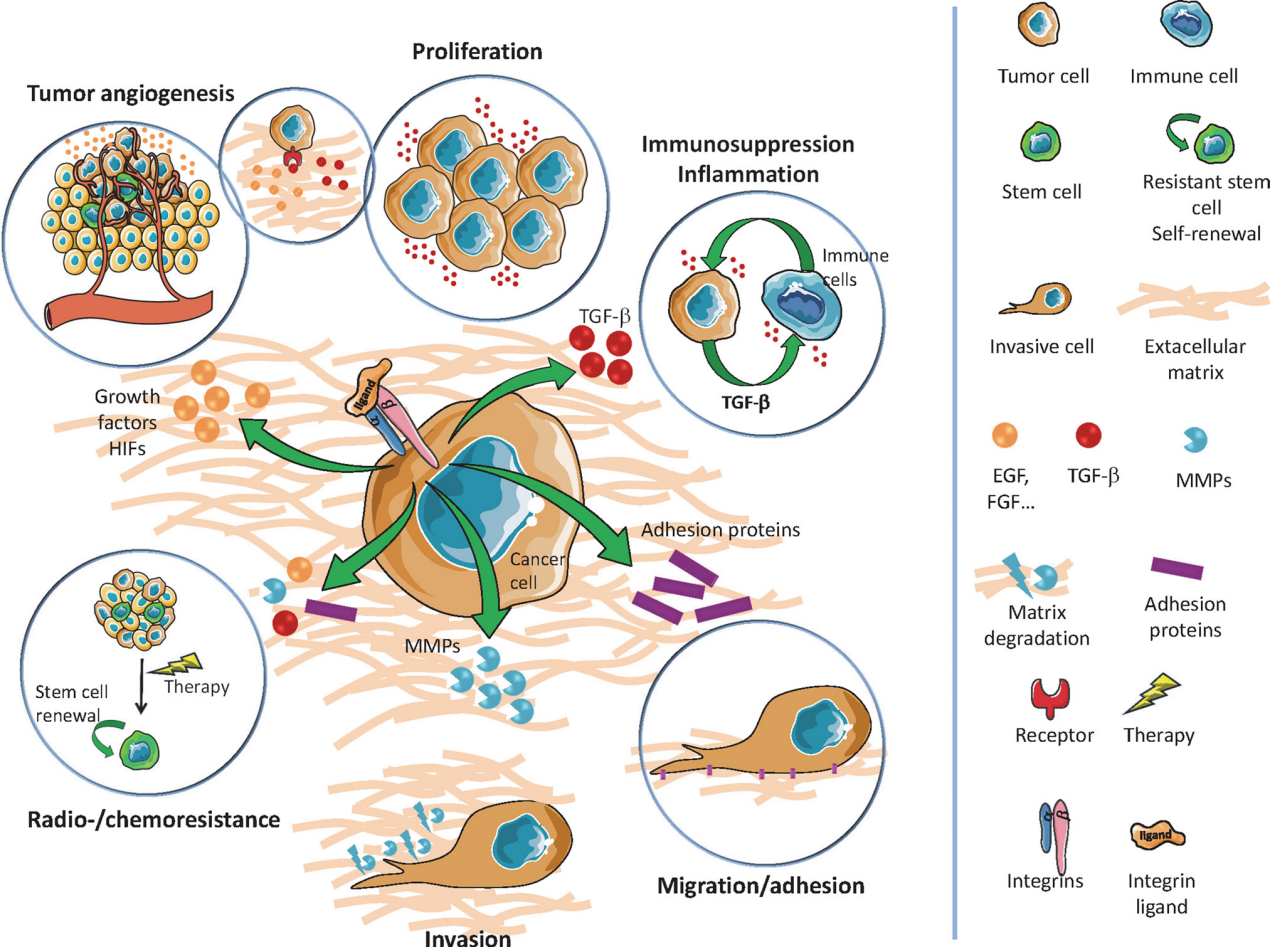
Overview of integrin interactions with glioblastoma microenvironment and niches

Tumor microenvironment modifications contribute to cancer aggressiveness and recurrence. GB, characterized by a specific and tightly regulated microenvironment, are among the most vascularized and hypoxic tumors [19]. Cell membrane-expressed integrins can interact and modulate GB specific microenvironment via pro-migratory and pro-invasive properties (previously reviewed [24] and described above), hypoxic signalling/conditions, growth factor pathways, immune system and GIC maintenance within their niches. These mechanisms are detailed below and Figure 5.

Hypoxia occurs in growing tumors when distant or abnormal tumor vasculature cannot provide blood supply. In GB, oxygen concentration decreases to 0.1–2% (2–10% in healthy brain). Hypoxia-signalling pathway, via hypoxia inducible factors (HIF-1/HIF-2), is then activated to promote, among others, angiogenesis. In different tumor types, hypoxia could up-regulate integrins (α6, αvβ3), which contribute to hypoxia cell adaptation [122, 123]. In GB, integrins are part of this hypoxia-signalling pathway and strongly support GB angiogenesis. For instance, α6-overexpressing U87 cells form bigger tumors than wild-type U87 in immunodeficient mice due to improved vascularisation [37]. Moreover, EGFRvIII/β3 integrin complexes were identified as promoter of GB progression in hypoxic environment [124]. Additionally, our laboratory demonstrated that αvβ3/αvβ5 were overexpressed in hypoxic U87 and SF763 GB cells, leading to FAK activation and subsequent RhoB/GSK-3-dependent HIF-1 stabilization [125]. In this study, the αvβ3/αvβ5-inhibitor Cilengitide, known to induce tumor regression via angiogenesis blockade and tumor growth inhibition [68], significantly decreased *in vitro* HIF-1 expression under hypoxia. Additionally, siRNA-mediated β3 inhibition led *in vivo* to an oxygenation of U87 xenografts [125]. It was also demonstrated in U87 that the T3 thyroid hormone, through iodothyronine receptor domains on αvβ3 and a Src/PI3K/ERK pathway, could enhance cell proliferation and HIF-1 expression [126]. Finally, in U251 cells, melatonin, a potent antioxidant, was shown to reduce cell migration/invasion in hypoxia through oxidative stress modulation and inhibition of the αvβ3/FAK/Proline-rich tyrosine Kinase 2 pathway [127]. Concerning integrin-mediated downstream pathways, and particularly ILK, two small-molecule ILK inhibitors (QLT0254/QLT0267) led *in vitro* to U87 cell cycle arrest and apoptosis, associated with AKT inhibition and VEGF secretion. *In vivo*, QLT0267 reduced U87 tumor growth by downregulating HIF-1 and VEGF, suggesting that ILK contribute to GB angiogenic process [128]. Consequently, specific integrins and associated-downstream pathways participate to hypoxia signalling in GB and then could impact on tumor aggressiveness.

As previously described, integrins cooperate with growth factors and/or their receptors and these pathways extensively cross talk. We focus here on TGF-β, involved in multiple cancer processes (for review [111]). TGF-β is secreted in a latent form consisting of a complex of three proteins: TGF-β (isoforms 1/2/3), inhibitor LAPs (latency-associated protein) and ECM-binding protein LTBPs (latent TGF-β binding proteins). To be activated, TGF-β needs to be liberated from latent complex, e.g. via conformational change or proteolysis. Interestingly, LAPs of TGF-β1 and TGF-β3 contain an integrin-binding site (RGD, arginine-glycine-aspartic acid sequence) and RGD-binding integrins (αv family, particularly αvβ6/αvβ8) can activate latent TGF-β through direct binding [110]. TGF-β is often overexpressed in GB and associated with tumor initiation and progression, as it promotes proliferation, invasion, angiogenesis, stemness, resistance and immune suppression [111]. Notably, inhibition of TGF-β pathway, up-regulated in glioma after irradiation [129], radiosensitizes GB cells [130]. Regarding the specific signalling, TGF-β binds to its receptors TGF-β-R-I/II/III and activates the canonical (SMAD proteins) and non-canonical (MAPK, PI3K/Akt, NF-κB, RhoGTPases…) pathways [111]. A recent study also demonstrates a network between integrins, aryl hydrocarbon receptor and TGF-β [131]. So αv integrins, through TGF-β activation, can promote GB progression.

Finally, integrin involvement in microenvironment may also depend on immune response modulation. Indeed, integrins regulate the recruitment of myeloid cells [22] and lead to immunosuppression or inflammation in favor of tumor progression, as exemplified for α4β1 [132]. In GB, a proteomic secretome analysis highlighted that osteopontin (interacting with αvβ3/αvβ5/αvβ6/αvβ1) and lactadherin (interacting with αvβ3/αvβ5) are able to induce M2 microglia reprogramming via an integrin/FAK/PI3K pathway [133]. M2 tumor-associated macrophages are recruited through association of their αvβ3 integrins with Periostin to favor tumor growth [134]. Another mechanism of integrin-mediated immunosuppression could involve TGF-β activation [110]. Depending on tumor context, GB or microglial cells-secreted TGF-β mediates immunosuppression either via the inhibition of natural killer cells, the IL-2R-mediated downregulation of proliferative signals in human T cells or the generation of immunosuppressive regulatory T cells [111].

Furthermore, all these microenvironment-related mechanisms can be transposed to GIC niches. GIC subpopulation is enriched in perivascular and hypoxic niches, which support their stemness and protect them from chemo/radiotherapies (for reviews [7, 19]). Moreover, hypoxia, HIFs and HIF-regulated genes play key role in GIC self-renewal and differentiation capacities compared to normal neural progenitors but also in GB cell dedifferentiation into GIC [9, 135]. Consequently, by modulating hypoxia and HIFs, integrins might regulate GIC characteristics, GIC differentiation and GB cell dedifferentiation. Additionally, integrins were previously described as key regulators to maintain stemness (see above) [48, 71]. Stem cell niches are indeed critical to preserve stemness, and integrins, by interacting with this particular local microenvironment, may provide signals allowing stem cells maintenance, depending or not on ECM interactions. Additionally, integrins could also enhance GIC properties via cooperation with growth factor receptors [24] and modulation of tumor immunity [12].

### Integrin-targeting in glioblastoma

Integrins, localized at the cell surface, represent attractive targets for GB treatment. Some integrins, like αvβ5, seems also quite selective to tumor cells, then limiting toxicity on healthy tissues. Diverse integrin-targeting agents (antibodies, peptidic/peptidomimetic antagonists and other small molecules) were therefore placed into clinical development and clinical trials, previously reviewed [23, 30] and updated in Table 1, were then set-up in different tumor types, including GB.

**Table 1:** Integrins targeting agents in cancer, including GB

MOLECULE	COMMERCIAL NAME	COMPANY	TYPE	TARGET	DISEASE	TRIAL STATUS	REFERENCES
GLPG0187	x	Galapagos SASU (France)	Integrin receptor antagonist	Broad spectrum integrins (αvβ1, αvβ3, αvβ5, αvβ6, αvβ8 and α5β1)	Advanced solid tumours	Phase I	[141]
EMD121974	Cilengitide	EMD pharm. Merck KGaA (Germany)	Cyclicized RGD- containing pentapeptide peptidomimetic	αvβ3 and αvβ5	Renal cell carcinoma, colon cancer, GB, melanoma, refractory advanced solid tumours, AML	Phase III	[142]
MEDI-522	Etaracizumab Abegrin	Medimmune Inc. (USA)	IgG1 humanized monoclonal antibody	αvβ3	Melanoma, prostate/colon/ thyroid cancer	Phase II	[143]
M200	Volociximab	Protein design labs (USA)	Chimeric monoclonal antibody	α5β1	Renal cell carcinoma, melanoma, NSCLC, pancreatic cancer	Phase II	[144]
PF0460541	x	Pfizer (USA)	IgG1 humanized monoclonal antibody	α5β1	Solid tumours	Phase I	[145]
ATN-161	x	Attenuon, LLC (USA)	Fibronectin like pentapeptide peptidomimetic	α5β1	Advanced solid tumours	Phases I/II	[146]
CNTO 95	Intetumumab	Centocor (USA)	Humanized monoclonal antibody	αv	Refractory advanced solid tumours	Phase II	[147]
EMD525797 DI-17E6	x	Merk (Germany)	Humanized monoclonal antibody	αv	Colorectal carcinoma and prostate	Phase II	[148]
E7820	x	Eisai medical research (USA)	Aromatic sulfonamide derivative	α2	Colorectal carcinoma	Phase II	[149]
OS2966	x	Oncosynergy (USA)	monoclonal antibody	β1	GB Ovarian cancer	Phase I	Planned in 2017 [150]

αvβ3/αvβ5, the most studied integrins in GB, are involved in tumor progression and angiogenesis. The main developed molecule to target αvβ3/αvβ5 is Cilengitide (EMD121974). This cyclized RGD pentapeptide potently blocks αvβ3/αvβ5 activation and preclinical studies demonstrated its efficacy *in vitro* and *in vivo* in GB (for review [68]). Consequently, this molecule entered into phase I/II clinical trials in GB, in association with radio/chemotherapies, first in recurrent and then in newly diagnosed GB. These trials, previously reviewed [68] and updated in Table 2, showed interesting clinical responses with a good tolerance and clinical benefice on progression free survival (PFS) as well as on OS (Table 2). The CENTRIC (phase III) and CORE (phase II) clinical trials were then designed to evaluate Cilengitide efficacy in GB patients with methylated or unmethylated MGMT gene promoter, respectively. However, these studies failed to achieve expected results, with no OS improvement (Table 2). Several reasons were evoked and notably the fact that Cilengitide short half-life could lead to a weak systemic concentration and an additional difficulty to reach its targets [136]. The absence of an adapted biomarker to identify responding GB patients was also mentioned. A retrospective study showed that αvβ3/αvβ5 expression did not correlate with an improved OS in CENTRIC patients and that only an overexpressed αvβ3 in tumor cells, but not endothelial cells, of CORE cohort patients may be associated to a better outcome in Cilengitide-treated arm (Table 2). Moreover, GIC, the most resistant cells within the tumoral mass, express low αvβ3/αvβ5 levels [35]. So, it could be hypothesized that Cilengitide could not conveniently target GIC, one of the major reservoirs for GB recurrence. However, even if Cilengitide did not improve OS and will not be further developed as an anticancer drug, integrins still remains under consideration in GB and other solid tumors (Table 1).

**Table 2:** Summary of clinical trials using cilengitide in GB

TRIAL NAME	PHASE	PATIENTS	TUMOR TYPE	CILENGITIDE DOSE + TREATMENTS	OUTCOMES (OS in months, PFS at 6 months)	REFERENCES
NABTT9911 NCT00006093	I	51	Recurrent malignant glioma	Single agent 120 to 2400 mg/m^2^ 2×/week	OS: 5.6	[151]
PBTC-012 NCT00063973	I	31	Recurrent pediatric brain tumours	Single agent 120 to 2400 mg/m^2^ 2×/week	Complete response: 1 Stable disease: 2	[152]
NCT00979862	I	45	Recurrent GB	Cilengitide 2000 mg/m^2^ 2×/week + Cediranib 30 mg daily	OS : 6.5 PFS-6 : 4.4 %	[153]
CILENT-0902 NCT01165333	I	40	Children With Diffuse Intrinsic Pontine Glioma	Radiotherapy + Cilengitide 240 to 1800 mg/m^2^	Completed	No published results
EMD-009 NCT00093964	II	81	Recurrent GB	Single agent 500 or 2000 mg/m^2^ 2×/week	500mg: OS: 6.5; PFS-6: 10% 2000mg: OS: 9.9; PFS-6: 15%	[154]
EMD-010	I/IIa	52	Newly diagnosed GB	Standard treatment + 500 mg/m^2^ 2×/week	OS : 16.1 PFS-6 : 69%	[155]
NABTC03-02 NCT00112866	II	30	Recurrent GB	Single agent 500 vs 2000 mg/m^2^ 3 days before surgery and then 2000 mg/m^2^ 2×/week	PFS-6: 12%	[156]
NABTT0306 NCT00085254	II	112	Newly diagnosed GB	Standard treatment + 500 vs 2000 mg/m^2^ 2×/week	500mg: OS: 17.4 2000mg: OS : 20.8	[157]
ACNS0621 NCT00679354	II	30	Recurrent or refractory pediatric brain tumours	Single agent 1800 mg/m^2^ 2×/week	Stable disease: 1	[158]
CORE NCT00813943	II	264	Newly diagnosed GB (unmethylated MGMT)	Standard treatment + 2000 mg/m^2^ 2 or 5×/week vs standard treatment	Cilengitide 2x/wk : OS : 16.3 Cilengitide 5x/wk : OS : 14.5	[50, 159]
EXCENTRIC NCT01124240	II	48	Newly diagnosed GB (unmethylated MGMT)	Standard treatment + Cilengitide 2000 mg/m^2^ 2×/week + Procarbazine 50 or 100 mg daily for 6 weeks (+ adjuvant treatment)	OS : 14.5 Median PFS : 7.4 months	[160]
CENTRIC NCT00689221	III	504	Newly diagnosed GB (methylated MGMT)	Standard treatment + 2000 mg/m^2^ 2×/week vs standard treatment	OS : 26.3	[33]
NCT01517776	II		Recurrent High-grade Gliomas (children and adolescents)	TMZ 75 mg/m^2^/d + Cilengitide 1800 mg/m^2^ 2×/week	Terminated. (due to an altered benefit/risk assessment)	
NCT01044225	II		Newly Diagnosed GB (unmethylated MGMT)	Standard treatment + Cilengitide 2000 mg/m^2^ 2×/week vs Standard treatment + Cetuximab (initial dose of 400 mg/m^2^ and then 250 mg/m^2^ per week)	Terminated. (due to results of the phase III RTOG0525/NCT00304031 trial)	

Besides being attractive therapeutic targets, integrins may offer several other clinical perspectives. First, they represent interesting prognosis biomarkers. For example, αvβ3 and α3β1 high expression was associated with poor prognosis in GB patients [25, 43]. Second, their extracellular part could be recognized using non-invasive imaging systems. Such tools may be useful for diagnosis, for selection of responding patients and for the follow-up of anti-integrin treatment efficacy. Interestingly, 18F-fluciclatide-labeled αvβ3/αvβ5 integrins are able to provide clinical information and guide patient care in GB [137]. Other examples are DA364, a RGD-cyclic probe allowing GB detection by near-infrared fluorescence imaging [138], or a new ^131^I-labeled RGD-cyclic peptide dimer for αvβ3 imaging [139]. Third, considering a specific integrins pattern on tumor cells compared to normal cells, integrins targeting to deliver therapeutics seems another promising application in GB treatment. Indeed, they could serve as delivering target to specifically supply tumor cells with chemotherapy, immunotherapy, radionucleotide, gene therapy agents or even nanoparticles. This could improve treatment efficacy but also reduce toxicity. For example, an anti-αvβ3 monoclonal antibody (Abegrin) associated with a radioimmunotherapeutic agent reduce tumor volume of orthotopic GB cells [140].

## CONCLUSIONS

Due to their involvement in GB radio/chemoresistance and progression (Table 3), integrins appear of great interest in GB treatment, either as targeted therapies, drug-delivering vectors or diagnostic tools for tumor imaging. Considering the strong GB heterogeneity, future preclinical and clinical studies have to focus on the particular integrins expression pattern within GB microenvironment. It is indeed crucial to identify the integrin profiles of specific subpopulations, notably GIC, and to correlate them to cell radio/chemosensitivity. Another challenge is to establish specific integrin signatures for each molecular GB subtype. These research axes represent key steps towards treatment personalization in GB and outcome improvement.

**Table 3:** Summary of integrins involved in GB

INTEGRINS	ROLES IN GB	REFERENCES
αvβ3	Migration Invasion Angiogenesis Survival Therapy resistance Prognostic marker	[36, 65–67] [24, 79] [77, 80, 81] [86] [78, 94, 95] [25]
αvβ4	Proliferation Therapy resistance	[49] [90]
αvβ5	Migration, Invasion Angiogenesis Therapy resistance	[65, 66] [77] [94, 95]
αvβ8	Invasion Angiogenesis	[73] [74]
α3β1	Migration, Invasion Stemness Prognostic marker	[70, 71] [71] [43]
α5β1	Proliferation Migration Invasion Survival Therapy resistance	[62–64] [82] [45, 75] [75, 91] [42]
α6β1	Invasion Stemness	[37, 69] [48]
α9β1	Migration Angiogenesis	[72] [83]

## SUPPLEMENTARY MATERIALS FIGURES AND TABLES


