# Supplementary material for: Interest of integrins targeting in glioblastoma according to tumor heterogeneity and cancer stem cell paradigm: an update
Source: Oncotarget. 2017 Aug 21;8(49):86947–68. doi: 10.18632/oncotarget.20372 (PMC5689739; doi:10.18632/oncotarget.20372)
Supplement: Supplementary file 1 [file oncotarget-08-86947-s001.pdf]

Interest of integrins targeting in glioblastoma according to tumor heterogeneity and cancer stem cell paradigm: an update

SUPPLEMENTARY MATERIALS

Molecular Subtype (n= 500)	
Classical	138 (27.6%)
Mesenchymal	150 (30.0%)
Neural	77 (15.4%)
Proneural	125 (25.0%)
Normal	10 (2.0%)

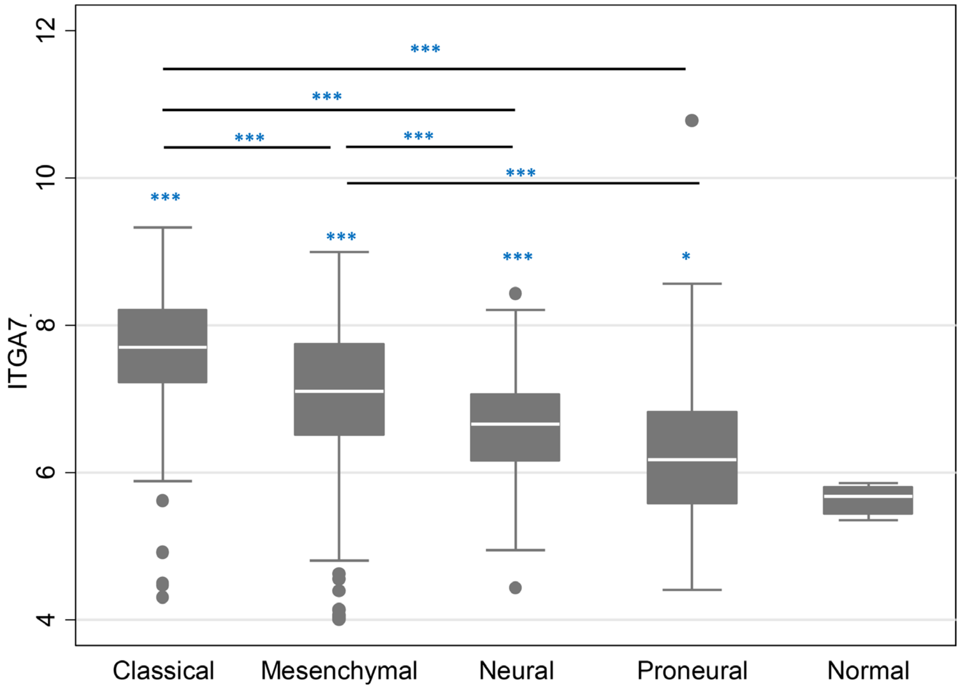

Supplementary Figure 1: Expression of *ITGA7* in TCGA GB samples according to their molecular subtypes. Plots were established using TCGA Affymetrix dataset ($n = 500$). $*p \leq 0.05$; $**p \leq 0.01$; $***p \leq 0.001$. When not stated, values are compared to the "normal" subgroup.

Molecular Subtype(n= 149)	
Classical	37 (24.8%)
Mesenchymal	47 (31.5%)
Neural	26 (17.4%)
Proneural	34 (22.8%)
Normal	5 (3.4%)

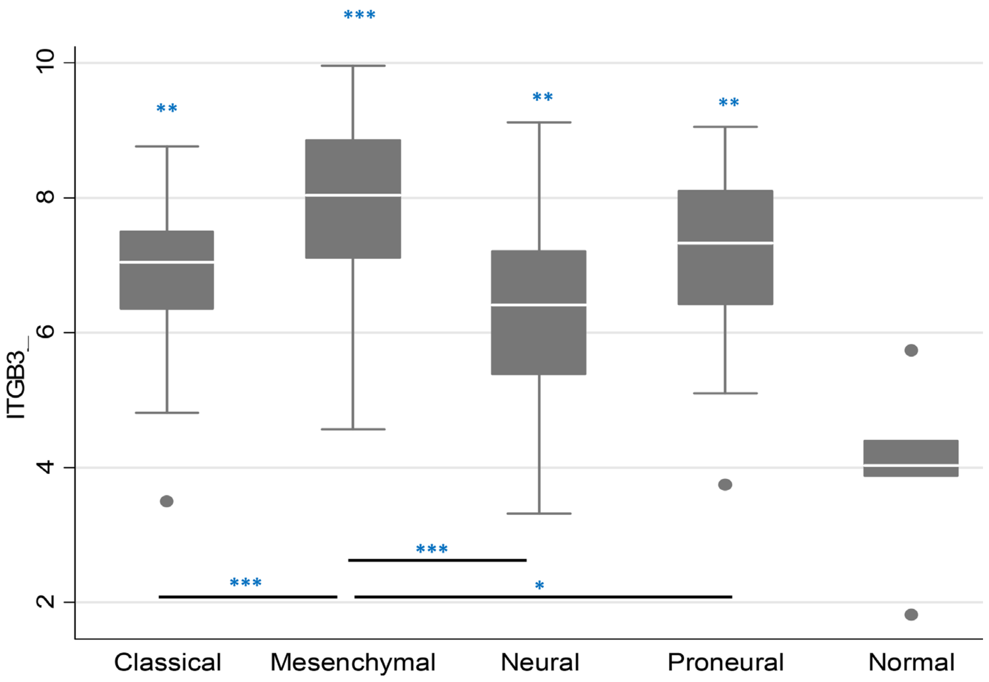

Supplementary Figure 2: Expression of *ITGB3* in TCGA GB samples according to their molecular subtypes. Expression plots were established using TCGA Illumina dataset, $n = 149$. *$p \leq 0.05$; **$p \leq 0.01$; ***$p \leq 0.001$. When not stated, values are compared to the "normal" subgroup.

Supplementary Table 1: Expression of main integrins in TCGA GB samples in comparison with control brain tissues

A

	**Solid Tissue Normal** **N= 10**	**Primary Tumor** **N= 490**	
ITGB1 (n= 500)			**p < 0.0001**
Median	5.4	5.8	
(Range)	(5.2: 5.5)	(5.3: 6.4)	
ITGB3 (n= 500)			p = 0.5835
Median	4.2	4.2	
(Range)	(4.2: 4.3)	(3.9: 6.0)	
ITGB5 (n= 500)			**p < 0.0001**
Median	5.6	6.6	
(Range)	(5.4: 5.8)	(4.6: 8.8)	
ITGB8 (n= 500)			**p < 0.0001**
Median	4.4	5.3	
(Range)	(4.0: 4.8)	(3.8: 7.5)	
ITGA5 (n= 500)			**p < 0.0001**
Median	5.4	7.1	
(Range)	(5.0: 6.2)	(4.9: 10.3)	
ITGA6 (n= 500			**p = 0.0004**
Median	6.3	7.1	
(Range)	(6.0: 6.8)	(4.2: 10.4)	
ITGA3 (n= 500)			**p = 0.0097**
Median	5.0	5.4	
(Range)	(4.7: 5.4)	(4.1: 9.8)	
ITGAV (n= 500)			**p < 0.0001**
Median	8.8	10.4	
(Range)	(8.4: 9.1)	(6.5: 12.5)	
ITGB4 (n= 500)			**p = 0.0008**
Median	4.5	4.9	
(Range)	(4.4: 4.7)	(4.1: 7.1)	
ITGA7 (n= 500)			**p < 0.0001**
Median	5.7	7.0	
(Range)	(5.4: 5.9)	(4.0: 10.8)	

B

	Solid Tissue Normal<br>*N*= 5	Primary Tumor<br>*N*= 144	
ITGB1 (*n*= 149)			***p* = 0.0006**
Median	10.2	12.2	
(Range)	(9.8: 11.8)	(10.8: 14.3)	
ITGB3 (*n*= 149)			***p* = 0.0005**
Median	4.0	7.3	
(Range)	(1.8: 5.7)	(3.3: 10.0)	
ITGB5 (*n*= 149)			***p* = 0.0029**
Median	9.5	10.7	
(Range)	(9.3: 9.7)	(8.5: 12.5)	
ITGB8 (*n*= 149)			***p* = 0.0030**
Median	10.7	12.2	
(Range)	(10.3: 11.6)	(7.7: 14.2)	
ITGA5 (*n*= 149)			***p* = 0.0002**
Median	7.6	10.6	
(Range)	(6.9: 8.7)	(7.9: 13.2)	
ITGA6 (*n*= 149)			*p = 0.0551*
Median	9.8	10.6	
(Range)	(9.5: 10.7)	(8.7: 12.5)	
ITGA3 (*n*= 149)			*p = 0.0592*
Median	9.5	10.3	
(Range)	(8.1: 9.7)	(5.6: 14.1)	
ITGAV (*n*= 149)			***p* = 0.0019**
Median	11.4	12.4	
(Range)	(11.1: 12.1)	(10.7: 14.2)	
ITGB4 (*n*= 149)			*p* = 0.2295
Median	10.3	10.9	
(Range)	(9.4: 11.2)	(6.5: 13.8)	
ITGA7 (*n*= 149)			***p* = 0.0044**
Median	10.3	11.6	
(Range)	(10.2: 10.4)	(8.0: 14.5)	

Median were established using TCGA Affymetrix (1A) and Illumina (1B) datasets, $n = 500$ and $n = 149$, respectively. *p*-values are given between all GB samples and to the “normal” subgroup.

SUPPLEMENTARY MATERIALS AND METHODS

TCGA databanks and related statistical analyses

We used The Cancer Genome Atlas (TCGA) datasets to analyze integrins gene expression in newly diagnosed primary GB patients [39, 49]. For gene expression, two datasets (processed data : version 2015-02-24, downloaded from https://genome-cancer.ucsc.edu) were used based either on microarray data (Affymetrix HT Human Genome U133a microarray platform by the Broad Institute of MIT and Harvard University, $n = 500$) or RNA-seq data (Illumina HiSeq 2000 RNA Sequencing platform by the University of North Carolina TCGA genome characterization center, $n = 149$). We also assessed OS using the TCGA Affymetrix dataset among a subgroup of newly diagnosed primary GB patients treated by standard chemo-radiotherapy ($n = 184$). For this OS analyses, gene expression levels were dichotomized based on a high expression cutoff (within the 75% quartile).

Multiple comparisons between groups were performed using Mann–Whitney U test. The Benjamini-Hochberg procedure was applied for multiple comparisons (Figure 4; Supplementary Figures 1 and 2). All survival rates were estimated by the Kaplan-Meier method with 95% confidence intervals (CI). Univariate analyses were performed using Cox proportional hazard model. Multivariate analyses were then performed using Cox proportional hazard model, including prognostic clinical covariates with a p-value < 0.10 in univariate analysis (Age, G-CIMP status and Karnofsky score). All reported p-values were two-sided. For all statistical tests, differences were considered significant at the 5% level. Statistical analysis was performed using STATA 12 software.
